# Effectiveness of a Home-Based Video-Guided Hip Muscle Exercise Program in Elderly Patients Following Bipolar Hemiarthroplasty for Femoral Neck Fracture: A Randomized Controlled Trial

**DOI:** 10.7759/cureus.112184

**Published:** 2026-07-06

**Authors:** Chawarat Sripon, Atiporn Therdyothin, Tanawat Amphansap, Noppavut Sirirak, Nacharin Phiphopthatsanee, Chinundorn Putananon, Wasin Wichitpreeda, Withawat Jaderojananont

**Affiliations:** 1 Department of Orthopedics, Police General Hospital, Bangkok, THA; 2 Psychiatry, Faculty of Medicine, King Mongkut's Institute of Technology Ladkrabang, Bangkok, THA

**Keywords:** femoral neck fracture, harris hip score, hip muscle strength, home based rehabilitation program, rehabilitation

## Abstract

Background: Physical therapy after a femoral neck fracture is essential but is limited by poor compliance and incorrect exercise. To improve this, we developed the home-based video-guided music program (HBW-MV) program. We hypothesize that this program will improve functional outcome.

Study design and methods: This was a randomized controlled trial from 2023 to 2024 on elderly patients with cementless bipolar hemiarthroplasty after femoral neck fracture. Thirty patients were equally allocated into the intervention group and the control group. Both received pre-discharge physical therapy instructions from an orthopedic trainee. Functional outcomes were assessed using Harris Hip Score (HHS) from two to 12 weeks. Hip strength was measured by a handheld dynamometer. Anxiety and pain were assessed using a visual analog scale. Compliance was assessed by a record table.

Results: Both groups showed an increase in HHS at two, six, and 12 weeks postoperatively. The intervention group demonstrated significantly higher HHS compared with the control group at all time points, with mean scores of 49.40±6.10, 65.20±6.09, and 82.30±5.85 versus 39.73±7.80, 53.53±5.84, and 67.67±6.33, respectively (p<0.05). At the 12-week follow-up, significantly higher hip muscle strength in abduction, adduction, and flexion was observed in the intervention group relative to the control group (p < 0.05), whereas hip extension strength, although improved in both groups, did not show a statistically significant between-group difference. At both six and 12 weeks postoperatively, anxiety scores were significantly reduced in the intervention group relative to the control group (six weeks: 2.87±0.91 vs 3.47±0.64, p=0.023; 12 weeks: 1.47±0.51 vs 2.27±0.96, p=0.004). Postoperative pain scores were also significantly lower in the intervention group at 12 weeks (1.20±0.41 vs 1.93±0.88, p=0.004). Patient compliance with the rehabilitation program was significantly higher in the intervention group at six and 12 weeks.

Conclusions: This research shows the HBW-MV program significantly improved functional outcomes, hip strength, and compliance. Additionally, it reduced anxiety and postoperative pain effectively.

## Introduction

Among aging populations, femoral neck fractures are a common and serious condition that imposes a substantial burden in terms of morbidity, mortality, and healthcare costs on a global scale [1,2]. In Thailand, the incidence of hip fractures has continued to increase in parallel with population aging, leading to a growing burden on patients, caregivers, and healthcare systems [3,4]. Elderly patients sustaining femoral neck fractures often experience significant functional decline, loss of independence, and increased mortality even after surgical treatment [5,6].

Bipolar hemiarthroplasty is widely accepted as a standard surgical option for displaced femoral neck fractures in elderly patients, providing reliable pain relief and allowing early mobilization [7]. However, surgical treatment alone is insufficient to ensure optimal functional recovery. Postoperative physical therapy plays a crucial role in restoring hip function, muscle strength, balance, and ambulation, thereby reducing long-term disability and fall risk [8-10].

Despite its importance, adherence to conventional outpatient physical therapy among elderly patients remains suboptimal. Common barriers include postoperative pain, anxiety, transportation difficulties, limited access to rehabilitation services, and inadequate supervision during home exercises [11-13]. Home-based rehabilitation programs have therefore been developed to reduce costs and improve accessibility while maintaining rehabilitation effectiveness [14-16].

Music therapy has been explored as an adjunctive intervention in postoperative settings, primarily for its potential to reduce pain, anxiety, and psychological distress, which are key factors influencing patient participation and compliance [17-19]. Mechanistically, music may enhance exercise adherence and recovery in older adults through several pathways. Listening to music during exercise reduces perceived effort and anxiety, likely through distraction and modulation of limbic system activity [17,18]. Music also modulates emotional responses and activates descending inhibitory pain pathways, thereby reducing pain perception during rehabilitation [17]. These analgesic and anxiolytic effects are particularly relevant for older adults recovering from surgery, as music has been shown to improve postoperative recovery and reduce psychological distress in this population [18]. 

In addition, the video component of a home-based video-guided music program (HBW-MV) program provides a visual demonstration of each exercise, allowing patients to observe and imitate correct movement patterns. This visual guidance may enhance motor learning, improve exercise accuracy, and reduce the risk of incorrect performance, particularly in older adults who may have difficulty understanding written or verbal instructions alone. Together, these mechanisms support the role of music and video guidance in improving compliance and recovery outcomes. Additionally, video-based exercise programs may enhance motor learning, exercise accuracy, and motivation through audiovisual guidance, particularly in older adults [20]. However, evidence regarding the effectiveness of combining music therapy with structured video-guided rehabilitation following bipolar hemiarthroplasty in elderly patients remains limited. 

The primary objective of this study was to determine whether the HBW‑MV program improves functional outcome measured by the Harris Hip Score (HHS) [21] at 12 weeks compared with standard physical therapy. Secondary objectives were to compare improvements in hip muscle strength, postoperative pain, anxiety, and rehabilitation compliance between the two groups. We hypothesized that the HBW‑MV program would result in superior functional outcomes, greater hip muscle strength, reduced postoperative pain and anxiety, and improved compliance.

## Materials and methods

Study design 

This study was designed as a randomized controlled trial involving elderly patients with femoral neck fractures who underwent cementless bipolar hemiarthroplasty between 2023 and 2024.

This study was registered in the Thai Clinical Trials Registry (TCTR20260409002).

Sample size calculation

Sample size estimation was performed for comparing two independent means, assuming a two-sided significance level of 0.05 and a beta error of 0.02, with equal allocation between groups. The calculation was based on the HHS, referencing a study by Wang et al. [16]. The required sample size was 10 participants per group, including the potential loss to follow-up.

Patient inclusion and exclusion criteria

**Figure 1:**
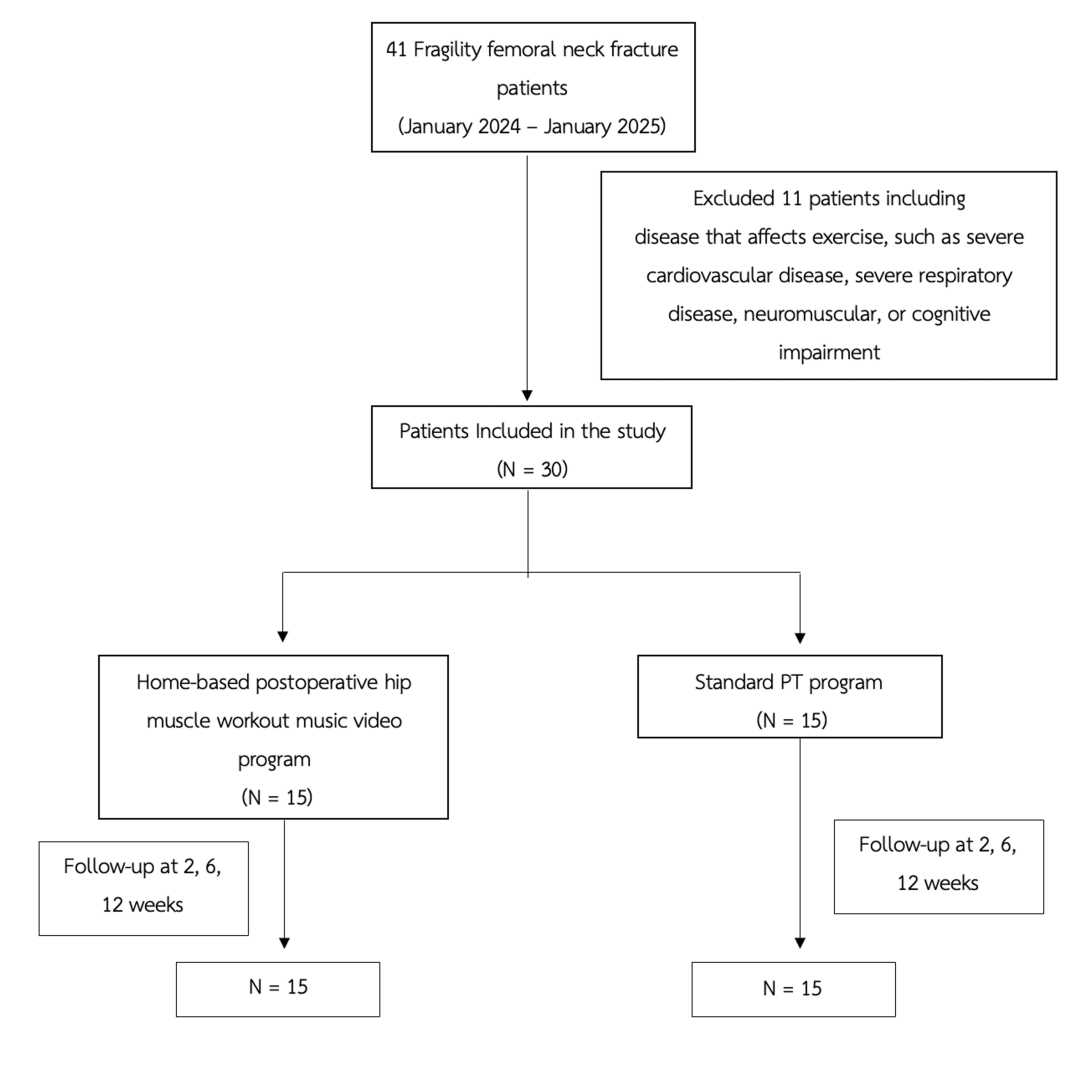
Study flow chart PT = physical therapy; HBW-MV = home-based video-guided music program; N = number of patients

**Figure 2:**
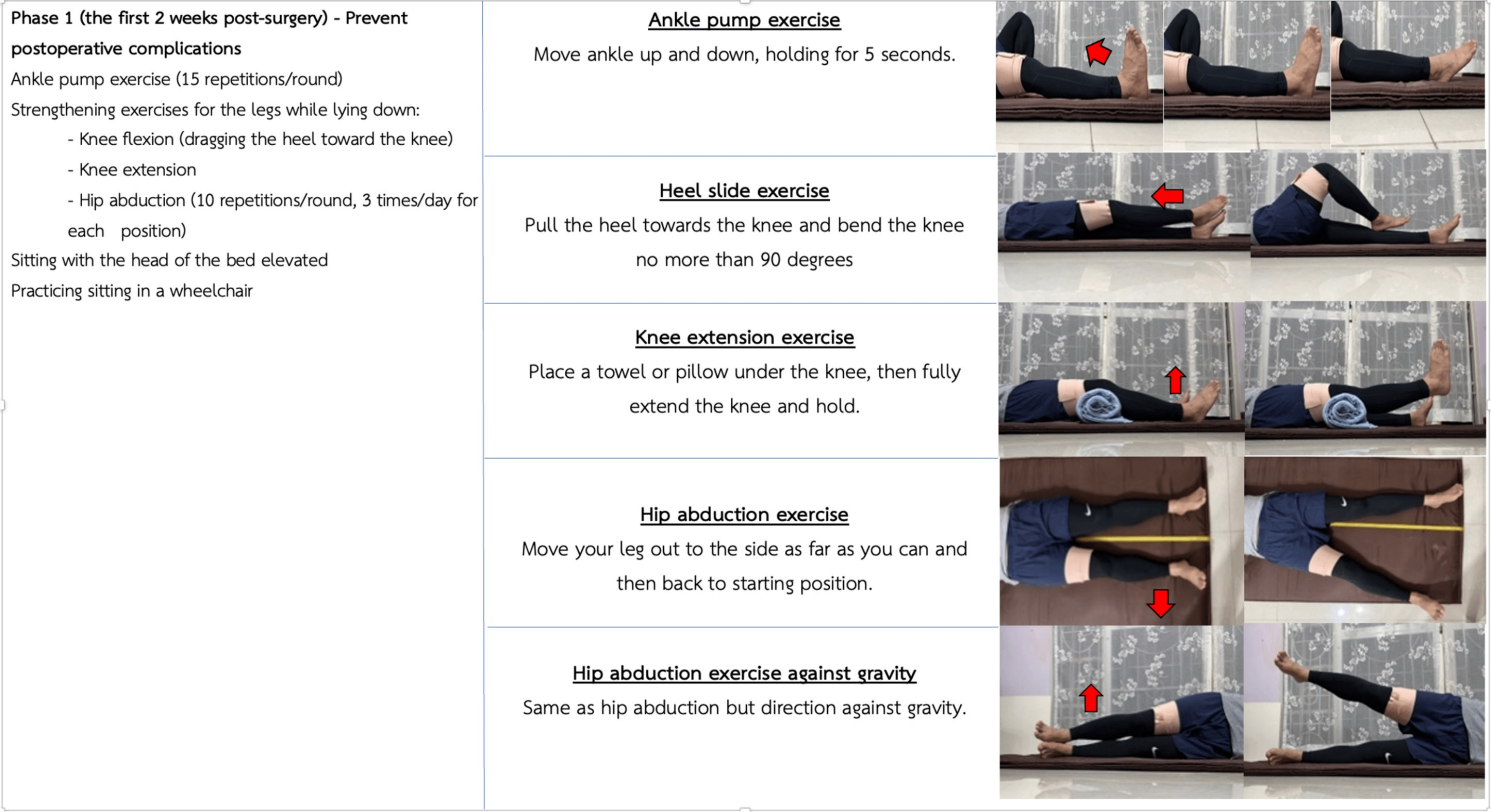
Rehabilitation program for patients with femoral neck fractures after bipolar hemiarthroplasty (Phase 1)

**Figure 3:**
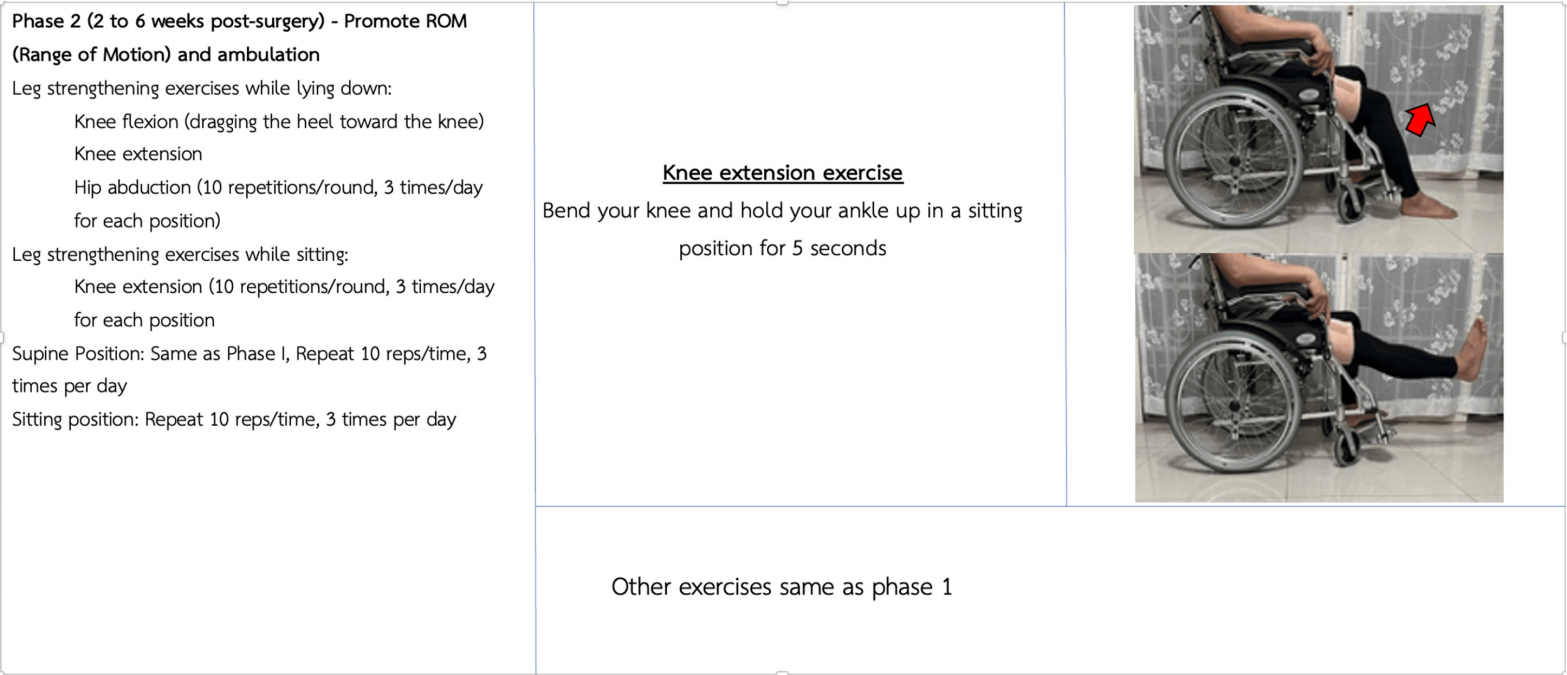
Rehabilitation program for patients with femoral neck fractures after bipolar hemiarthroplasty (Phase 2)

**Figure 4:**
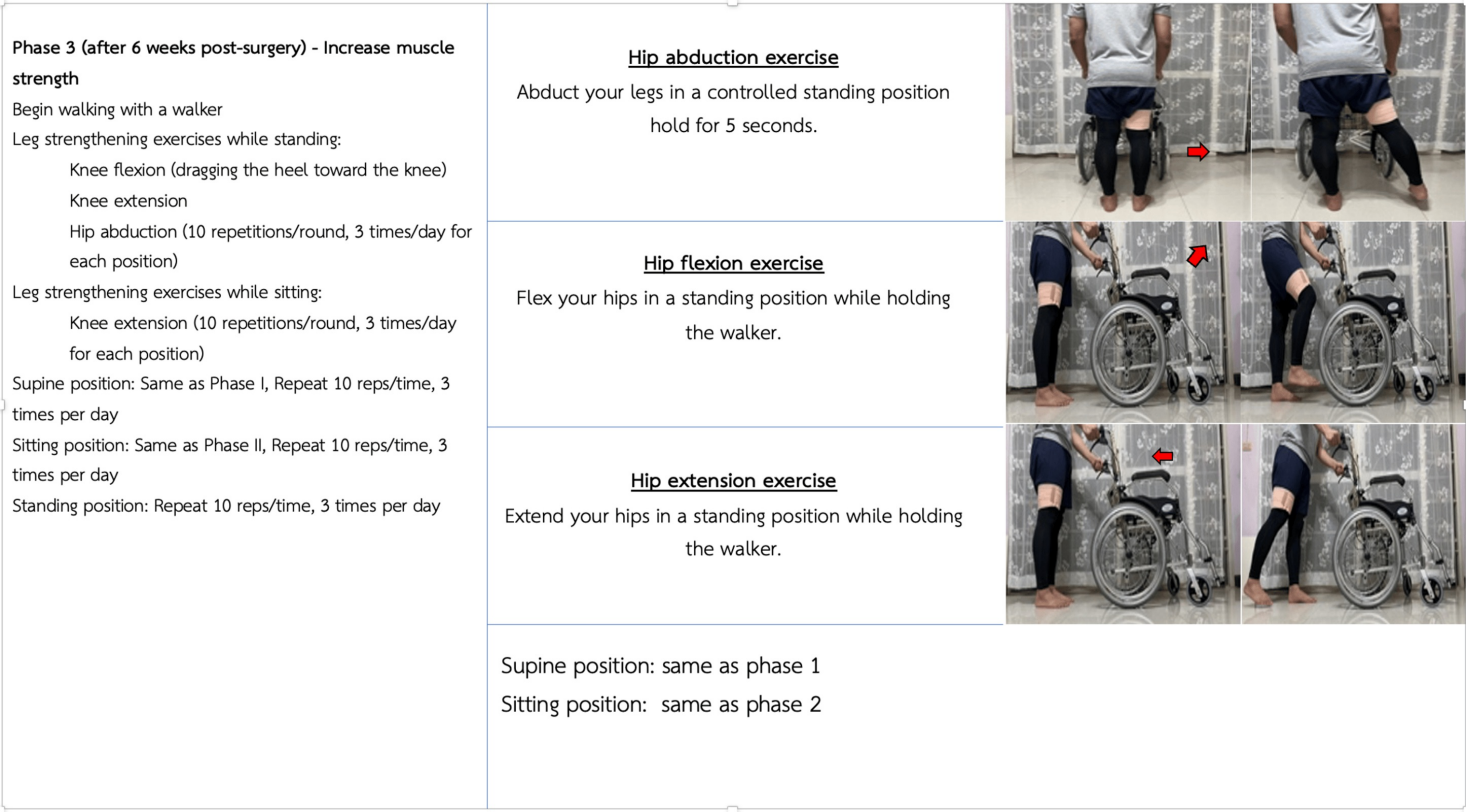
Rehabilitation program for patients with femoral neck fractures after bipolar hemiarthroplasty (Phase 3)

After approval from the Institutional Review Board (IRB 0068/2526), the study was conducted at Police General Hospital, Thailand. A total of 30 patients were enrolled, and the study design followed the applicable Consolidated Standards of Reporting Trials (CONSORT) 2010 guidelines (Figure 1). Patients aged more than 60 years who required treatment for primary femoral neck fracture and who underwent cementless bipolar hemiarthroplasty using an anterolateral approach were enrolled starting in January 2024. Included patients had to have had the ability to walk independently or walk with a gait aid for at least 10 meters prior to sustaining their fracture. Patients were excluded from this study if they had a disease that affects exercise, such as severe cardiovascular disease, severe respiratory disease, neuromuscular disease, psychiatric disease, dementia, or cognitive impairment. Patients with postoperative complications that adversely affect the ability to exercise were also excluded. Randomization was performed using a computer-generated sequence, and group allocation was concealed in sealed envelopes prior to assignment into the two rehabilitation groups: 15 patients for the HBW-MV program (intervention group) and 15 patients for standard physical therapy (control group). All patients were instructed on how to perform home-based physical therapy for femoral neck fracture by a physician before discharge from the hospital. The physical therapy program used standardized methods divided into three phases as detailed in Figures 2-4. 

The intervention group was given the HBW-MV program that taught exercises to patients and their relatives according to the instructional video for postoperative exercise. The video lasted five minutes. Patients performed the program three times daily. Each exercise consisted of 10 repetitions per cycle, and the whole set was repeated for three cycles, with a five‑second hold per movement, following the exercise table (Figures 2-4). The video file was saved directly on the patient’s smartphone or tablet. All patients were instructed on how to open and play the video and confirmed they could do so independently; therefore, caregiver assistance was not required. Before discharge, all patients received standardized face‑to‑face instruction on the prescribed exercises from the same orthopedic surgeon using a standardized script and checklist. Printed instruction sheets were provided to both groups. The intervention group additionally received the HBW-MV video file via Line (LY Corporation, Tokyo, Japan). The background music had a slow, steady rhythm (approximately 60 beats per minute, or one beat per second), corresponding to the prescribed five‑second movement cadence (five beats per repetition). This rhythmic cue was intended to help patients maintain correct movement timing while also enhancing engagement and motivation. The full video content and detailed exercise protocol are available from the corresponding author upon reasonable request.

In contrast, the control group received identical training in the form of instruction sheets to practice at home, without accompanying music videos. Both groups received a weekly standardized encouragement message via the Line application. A reminder phone call was made only if the logbook was not submitted for three consecutive days. The intervention group received the HBW-MV video file through Line. The control group received printed exercise instruction sheets (same exercises, dose, frequency) without the video.

Data collection 

Baseline information was obtained for all participants, including demographic characteristics, anthropometric measurements (body weight, height, and body mass index [BMI]), and clinical variables. Recorded clinical data comprised underlying comorbidities - such as hypertension, dyslipidemia, diabetes mellitus (type I or II), and chronic kidney disease stage III - as well as the American Society of Anesthesiologists (ASA) classification and pre-injury functional status.

Patients were scheduled for postoperative follow-up visits at two, six, and 12 weeks after surgery. At each follow-up visit, hip muscle strength, postoperative anxiety, postoperative pain, and treatment compliance were assessed. Functional outcome was defined as the primary outcome and was evaluated at all follow-up time points.

Functional outcome was assessed using HHS, which evaluates pain, functional activity, absence of deformity, and range of motion, with a total score ranging from 0 to 100. A score of 90-100 is considered a good functional outcome [21]. The HHS has been widely used to assess functional outcomes following hip surgery and has demonstrated acceptable validity and reliability [21]. In addition, the Thai version of the modified HHS has been shown to be valid and reliable for assessing functional outcomes in patients with hip abnormalities [22].

Hip muscle strength on the injured side was measured in abduction, adduction, flexion, and extension using a portable digital handheld dynamometer (wireless microFET2 Digital Dynamometer; Hoggan Scientific, Salt Lake City, UT, USA). Handheld dynamometry has demonstrated acceptable reliability for assessing lower extremity muscle strength in elderly populations [23].

Hip muscle strength was measured using a wireless microFET2 Digital Dynamometer. Patients were positioned as follows: for hip abduction, supine with leg abducted against resistance applied 5 cm above the lateral malleolus; for hip adduction, supine with leg adducted against resistance 5 cm above the medial malleolus; for hip flexion, supine with knee flexed 90 degrees, resistance applied 5 cm above the patella; for hip extension, prone with knee flexed 90 degrees, resistance applied 5 cm above the knee joint. For each movement, patients were instructed to exert maximal force and maintain for approximately five seconds. The test was repeated five times with a 30‑second rest between attempts, and the highest value (in Newtons) was recorded.

Postoperative anxiety and pain were assessed using a visual analog scale (VAS), a widely used and validated instrument for measuring subjective symptoms. Treatment compliance was evaluated by reviewing patient logbooks documenting the frequency of prescribed physical therapy exercises. Compliance (%) was calculated as (number of exercise sessions actually performed / total prescribed sessions) × 100.

Outcome assessors (those performing HHS, dynamometry, and VAS evaluations) and the data analyst were blinded to group allocation throughout the study period.

Statistical analysis 

Patient demographic and clinical characteristics were summarized using descriptive statistics. Continuous variables were expressed as mean ± standard deviation and categorical variables were presented as frequency and percentage. The chi-square test was used to compare gender, underlying disease, ASA classification, and pre-injury status. An independent t-test was used to compare the results of HHS, hip muscle strength, postoperative pain, and anxiety between groups, while a paired t-test was used to compare within-group changes over time. For compliance, the Mann‑Whitney U test was used (reported as Z‑value) due to non‑normal distribution. Because the sample size was small and follow‑up was complete with no missing data, independent t‑tests at each time point were used as the primary analysis for between‑group comparisons. Paired t‑tests were also used for within‑group comparisons over time, as specified in the statistical analysis section. Data analysis was performed using IBM SPSS Statistics software, version 29.0.1 (IBM Corp., Armonk, NY, USA). Statistical significance was defined as a p-value < 0.05.

## Results

Thirty patients were included and equally allocated to the HBW-MV intervention group and the standard physical therapy control group.

Patient demographic and clinical characteristics are shown in Table 1. The mean age of participants was 74.6±6.3 years and 22 patients (73%) were women. The average BMI was 22.4±2.0 kg/m2. No patient had postoperative complications, including infection, secondary fracture, or hip dislocation. 

**Table 1:** Baseline characteristics SD = standard deviation; BMI = body mass index; CKD = chronic kidney disease; ASA = American Society of Anesthesiologists

	Intervention group (N=15)	Control group (N=15)	χ² - value	t-value	p-value
			Age (years)	74.04±6.74		75.13±5.87	-	-0.47	0.324
Sex			0.00	-	1
Male	26.70%	26.70%			
Female	73.30%	73.30%			
Body weight (Kilogram)	61±7.64	58.13±6.11	-	1.13	0.133
Height (centimeter)	164.07±7.76	162.4±5.42	-	0.68	0.251
Body mass index (kg/m2)	22.81±2.36	22±1.78	-	1.08	0.461
Medical history					
Hypertension	86.07%	66.70%	1.67	-	0.195
Type II diabetes mellitus	60%	33.30%	2.14	-	0.143
Dyslipidemia	60%	66.70%	0.15	-	0.705
CKD 3-5	13.30%	13.30%	0.00	-	1
Functional status			1.54	-	0.464
ASA I	13.30%	26.70%			
ASA II	73.30%	53.30%			
ASA III	13.30%	20.00%			
Dominant side			2.14	-	0.143
Right	86.70%	100%			
Left	13.30%	0%			
Gait aid			0.54	-	0.464
Use	60.00%	46.70%			
Preinjury status			0.37	-	0.543
Active	6.70%	13.30%			
Household	93.30%	86.70%			
Fracture side			0.13	-	0.715
Right	53.30%	46.70%			
Left	46.70%	53.30%			

**Figure 5:**
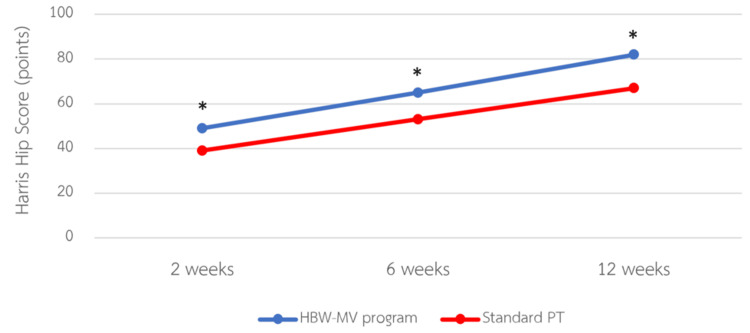
Postoperative improvement in HHS PT = physical therapy; HBW-MV = home-based video-guided music program; HHS = Harris Hip Score [21]; * = p-value < 0.05 between groups

After evaluating the outcome parameters, HHS improved from two to 12 weeks. The intervention group had HHS of 49.4±6.10, 65.2±6.09, and 82.3±5.85 at two, six, and 12 weeks, respectively, while the control group had HHS of 39.73±7.80, 53.53±5.84, and 67.67±6.33 at two, six, and 12 weeks. The intervention group shows statistically significant (p-value < 0.05) better HHS than the control group (Table 2, Figure 5). 

**Table 2:** Postoperative improvement in HHS SD = standard deviation; HHS = Harris Hip Score [21]; * = p-value <0.05

	Intervention group (N=15)	Control group (N=15)	t-value	p-value
2 weeks	49.4±6.10	39.73±7.80	3.78	<0.001*
6 weeks	65.2±6.09	53.53±5.84	5.49	<0.001*
12 weeks	82.3±5.85	67.67±6.33	6.63	<0.001*

**Figure 6:**
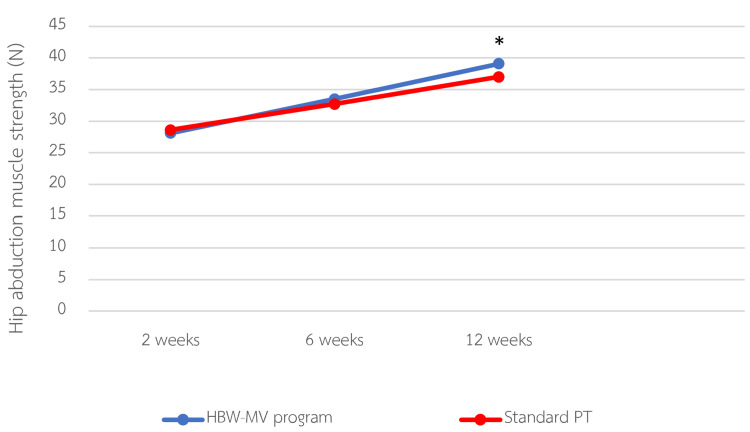
Postoperative improvement in hip abduction muscle strength (Newtons) PT = physical therapy; HBW-MV = home-based video-guided music program; * = p-value < 0.05 between groups

**Figure 7:**
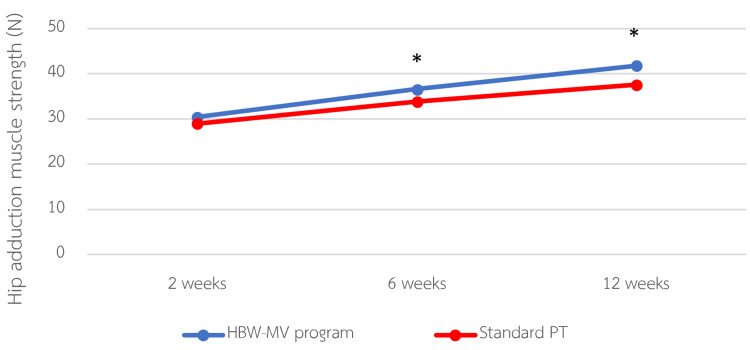
Postoperative improvement in hip adduction muscle strength (Newtons) PT = physical therapy; HBW-MV = home-based video-guided music program; * = p-value < 0.05 between groups

**Figure 8:**
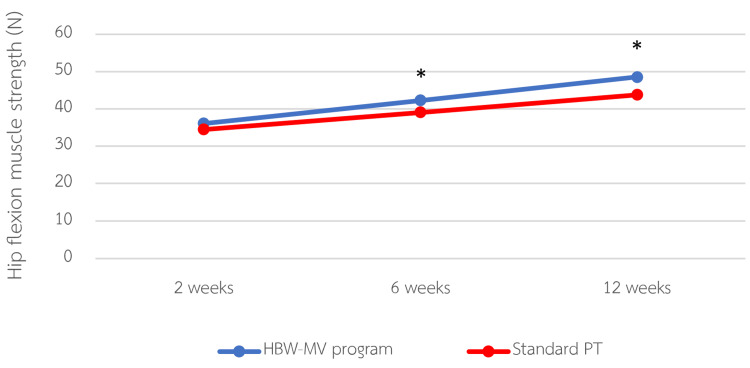
Postoperative improvement in hip flexion muscle strength (Newtons) PT = physical therapy; HBW-MV = home-based video-guided music program; * = p-value < 0.05 between groups

**Figure 9:**
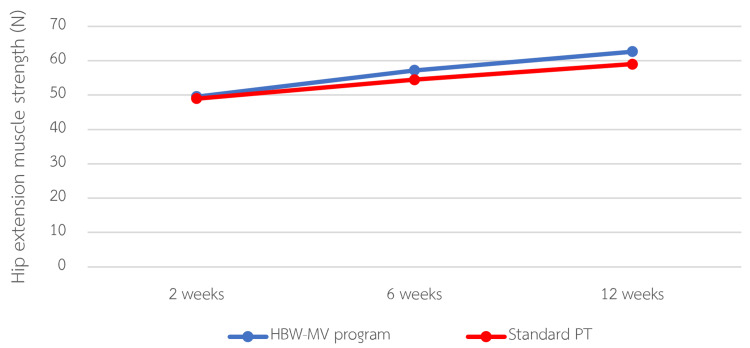
Postoperative improvement in hip extension muscle strength (Newtons) PT = physical therapy; HBW-MV = home-based video-guided music program

Hip muscle strength of the injured side (Newtons) improved from two to 12 weeks in both groups (Table 3). The intervention group demonstrated significantly greater hip abduction strength at 12 weeks compared with the control group (39.11±1.87 vs. 37.08±2.56; p-value = 0.01) (Figure 6). Additionally, hip adduction strength showed statistically significant differences at six weeks (36.66±2.97 vs. 33.80±4.21; p-value = 0.021) and at 12 weeks (41.82±2.48 vs. 37.62±4.51; p-value = 0.002) (Figure 7). Hip flexion strength also demonstrated statistically significant differences at six weeks (42.36±4.83 vs. 39.19±2.94; p-value = 0.019) and at 12 weeks (48.62±3.00 vs. 43.83±3.99; p-value < 0.001) (Figure 8). However, hip extension did not show any statistically significant difference at two, six, or 12 weeks (Figure 9). 

**Table 3:** Postoperative improvement in hip muscle strength (Newtons) * = p-value < 0.05

	Intervention group	Control group	t-value	p-value
	(N=15)	(N=15)		
Hip Abduction				
2 weeks	28.01±2.52	28.65±1.57	-0.81	0.238
6 weeks	33.59±1.99	32.70±1.91	1.24	0.113
12 weeks	39.11±1.87	37.08±2.56	2.45	0.010*
Hip Adduction				
2 weeks	30.45±2.68	28.92±3.66	1.29	0.102
6 weeks	36.66±2.97	33.80±4.21	2.15	0.021*
12 weeks	41.82±2.48	37.62±4.51	3.14	0.002*
Hip flexion				
2 weeks	36.17±5.45	34.59±3.11	0.96	0.17
6 weeks	42.36±4.83	39.19±2.94	2.18	0.019*
12 weeks	48.62±3.00	43.83±3.99	3.75	<0.001*
Hip extension				
2 weeks	49.65±5.47	49.01±10.13	0.21	0.416
6 weeks	57.25±5.20	54.49±10.12	0.94	0.178
12 weeks	62.72±4.39	59.07±10.89	1.21	0.119

Anxiety improved from two to 12 weeks in both groups. The anxiety scores were statistically significantly better in the intervention group compared to the control group at six weeks (2.87±0.91 vs. 3.47±0.64; p-value = 0.023) and at 12 weeks (1.47±0.51 vs. 2.27±0.96; p-value = 0.004), as shown in Table 4.

**Table 4:** Postoperative anxiety assessed by visual analog score (maximum score = 10). * = p-value < 0.05

	Intervention group (N=15)	Control group (N=15)	t-value	p-value
2 weeks	4.47±1.50	5.27±1.16	-1.63	0.057
6 weeks	2.87±0.91	3.47±0.640	-2.07	0.023*
12 weeks	1.47±0.51	2.27±0.96	-2.85	0.004*

Postoperative pain, also measured by a VAS, improved from two to 12 weeks in both groups. A statistically significant reduction in pain scores was observed in the intervention group relative to the control group at 12 weeks (1.20±0.41 vs. 1.93±0.88; p-value = 0.004), as shown in Table 5.

**Table 5:** Postoperative pain assessed by visual analog score (maximum score = 10). * = p-value < 0.05

	Intervention group (N=15)	Control group (N=15)	t-value	p-value
2 weeks	6.27±1.33	6.07±1.03	0.46	0.325
6 weeks	3.20±0.86	3.60±1.12	-1.10	0.141
12 weeks	1.20±0.41	1.93±0.88	-2.91	0.004*

Compliance, measured as a percentage, improved from two to 12 weeks in both groups. Compliance scores were statistically significantly better in the intervention group compared to the control group at six weeks (47.76±4.07 vs. 28.53±1.46; p-value < 0.001) and at 12 weeks (67.80±4.45 vs. 38.06±3.22; p-value < 0.001), as shown in Table 6.

**Table 6:** Compliance in performing physical therapy. * = p-value < 0.05

	Intervention group (N=15)	Control group (N=15)	z-value	p-value
2 weeks	25.52%±2.26%	22.46%±1.86%	1.43	0.153
6 weeks	47.76%±4.07%	28.53%±1.46%	4.28	<0.001*
12 weeks	67.80%±4.45%	38.06%±3.22%	4.52	<0.001*

## Discussion

Bipolar hemiarthroplasty remains a standard surgical treatment for displaced femoral neck fractures in elderly patients, with postoperative rehabilitation being a key determinant of functional recovery [7,8]. In this randomized controlled trial, patients who participated in the HBW-MV program demonstrated significantly better functional outcomes as measured by HHS compared with those receiving standard physical therapy alone. These improvements were observed consistently across all time points, suggesting that a structured home-based audiovisual rehabilitation approach may enhance early postoperative recovery.

Improvements in functional outcomes were accompanied by greater gains in hip muscle strength in the HBW-MV group. Hip muscle weakness and impaired balance are well-recognized contributors to gait instability and increased fall risk following hip fracture surgery [9-11]. In the present study, the HBW-MV group showed significantly greater improvements in hip abduction, adduction, and flexion strength compared with the control group. Among these, hip abductor strength is critical for pelvic stabilization and mediolateral balance during gait in older adults [10]. Although hip extension strength improved in both groups, no statistically significant between-group difference was observed. This finding may be explained by the limited activation of hip extensors during early postoperative ambulation with assistive devices and the relatively short follow-up period, which may have been insufficient to capture meaningful differences in extensor strength recovery [11,12].

The greater improvements observed in the HBW-MV group are consistent with prior evidence indicating that video-delivered exercise programs were associated with improvements in physical performance, including lower-limb strength, balance, and mobility in adults aged 60 years and older. Compared with text-based interventions such as leaflets, video-based guidance provides a richer multisensory experience through visual demonstration, auditory instruction, and motivating background music. These features can promote better understanding, engagement, and adherence [24]. This advantage may be explained by the cognitive theory of multimedia learning proposed by Richard E. Mayer, which emphasizes that combining visual and verbal inputs enhances understanding by enabling learners to form integrated mental representations [25].

Beyond motor learning, the integration of music may also have contributed to improved outcomes by modulating pain and anxiety. Music therapy has been increasingly studied as an adjunct to rehabilitation, particularly for its analgesic and anxiolytic effects. A systematic review by Cepeda et al. demonstrated that music listening could reduce pain intensity and analgesic requirements, potentially through distraction, emotional modulation, and activation of descending inhibitory pain pathways, although the overall clinical effect size was modest [17]. From a neurophysiological perspective, music may reduce sympathetic nervous system activity and modulate limbic system responses, thereby decreasing pain perception and anxiety during exercise [18].

Previous studies evaluating music therapy combined with physical therapy have yielded mixed results. Leonard reported no significant reduction in pain or exercise adherence when live music therapy was combined with lower extremity exercise after arthroplasty; however, the intervention duration was brief, and the exercise modality was not tailored to elderly postoperative patients [19]. In contrast, Laframboise-Otto et al. demonstrated that self-selected music administered for longer durations significantly reduced postoperative pain and distress following arthroplasty, although music was delivered independently rather than integrated into a structured rehabilitation program [20]. The HBW-MV program differs from these approaches by integrating low-tempo music directly with targeted hip muscle strengthening exercises delivered via video, which may enhance engagement, motor learning, and exercise accuracy.

Improved compliance observed in the HBW-MV group at six and 12 weeks is clinically meaningful, as pain and anxiety have been identified as major barriers to adherence in musculoskeletal rehabilitation, and adherence has also been reported as a persistent challenge in home-based rehabilitation following bipolar hemiarthroplasty [26,27]. By alleviating postoperative pain and anxiety, the HBW-MV program may indirectly enhance patient motivation and consistency in performing prescribed exercises, leading to better functional outcomes.

It is important to note that adherence and benefit may also be influenced by the patient’s pre-existing skills. Counterintuitively, patients with lower baseline skills may derive less benefit from demonstration videos than those who are already relatively proficient [28]. This phenomenon might be partly explained by challenge-threat appraisal theory, which posits that individuals will be more likely to perform the task if they perceive their skills as sufficient to meet the task demands [29]. In our study, all participants received physician-led instruction on performing home-based physical therapy for femoral neck fracture prior to enrolment. This preparatory education, along with simplicity of the exercise regimen, may increase perceived self-efficacy, thereby facilitating adherence.

This study has several strengths. To our knowledge, it is the first randomized controlled trial to evaluate a music-integrated, video-based hip muscle rehabilitation program specifically in elderly patients following bipolar hemiarthroplasty. A complete follow-up rate was achieved, and multiple clinically relevant outcomes were assessed, including functional scores, muscle strength, pain, anxiety, and compliance. No adverse events, recurrent fractures, dislocations, or falls were observed during the study period, supporting the safety of the intervention.

The observed differences are statistically significant and clinically meaningful. The mean HHS of the intervention group at 12 weeks (82.3) exceeded the threshold for a good outcome (≥80), and the between‑group difference of approximately 15 points surpassed the minimal clinically important difference (MCID) of 7-10 points reported for the HHS [21]. For hip muscle strength, the improvement in hip abduction strength (2.03 N) exceeded the standard error of measurement (SEM = 2 N) reported for handheld dynamometry [30], indicating that the observed change was beyond measurement error. The intervention group demonstrated better outcomes across all measured domains (HHS, muscle strength, pain, anxiety, and compliance). However, conclusions should be interpreted cautiously due to the small sample size, short follow‑up, single‑center design, self‑reported compliance, and inability to separate intervention components. Therefore, findings apply primarily to short‑term outcomes.

This study has several limitations that should be considered. The relatively small sample size and short follow-up duration of 12 weeks may limit the generalizability of the findings and preclude evaluation of long-term outcomes. The small sample size also limits statistical power; therefore, findings should be interpreted as preliminary and require further investigation in larger, multicenter studies. Another limitation is that our study design cannot separate the independent effect of music from that of video‑based instruction, because the control group received written instruction sheets rather than a video without music. Consequently, it is not possible to determine whether the observed benefits are attributable to the music, the video guidance, the structured exercise, or the combination of these components. Because the control group received written instructions rather than a video‑only or music‑only condition, the independent contributions of video guidance and music could not be isolated. Therefore, the observed benefits of the HBW‑MV program should be interpreted as the combined effect of music and video guidance, rather than the specific effect of music alone. Additionally, compliance monitoring through a mobile messaging application may have introduced a reminder effect, potentially inflating adherence rates. Future investigations should incorporate larger study populations, longer follow-up durations, and comparative analyses of various music interventions or delivery strategies to strengthen the evidence base.

## Conclusions

Postoperative physical therapy is an essential component of recovery in elderly patients with femoral neck fractures, aiming to reduce disability and promote functional restoration after surgery. This randomized controlled trial demonstrated that the HBW-MV program was associated with superior functional outcomes, greater improvement in hip muscle strength, higher rehabilitation compliance, and reduced postoperative pain and anxiety compared with conventional physical therapy following bipolar hemiarthroplasty. These findings suggest that integrating structured exercise with music-based video guidance may enhance early postoperative rehabilitation in elderly patients. Further studies with larger sample sizes and longer follow-up durations are warranted to confirm the long-term benefits of this intervention.
